# HPTLC Analysis, Antioxidant and Antigout Activity of Indian Plants

**Published:** 2014

**Authors:** Shivraj Hariram Nile, Se Won Park

**Affiliations:** Department of Molecular Biotechnology, College of Life and Environmental Sciences, Konkuk University, Seoul 143-701, Korea.

**Keywords:** HPTLC, Medicinal plants, Xanthine oxidase, Antioxidant, Flavonoids

## Abstract

The HPTLC analysis, antioxidant, and antigout activity of *Asparagus racemosus, Withania somnifera, Vitex negundo, Plumbago zeylanica, Butea monosperma *and *Tephrosia purpurea* extracts were investigated. The chemical fingerprinting were carried out by high performance thin layer chromatography (HPTLC), antioxidant activity by ABTS, DPPH, FRAP radical scavenging assays, and antiogout activity by cow milk xanthine oxidase. The HPTLC fingerprint qualitatively revealed predominant amount of flavonoids. The TEAC values ranged from 45.80 to 140 µM trolox/100 g dry weight for ABTS, from 85 to 430 µM trolox/ 100 g dw DPPH, and 185 to 560 µM trolox/100 g dw for FRAP respectively. Plants used in this study was found to inhibit the toxicity, as seen from the decreased LPO and increased GSH, SOD and CAT levels. The total phenolic and flavonoid content ranged from 10.21 to 28.17 and 5.80 to 10.1 mg of gallic acid equivalents (GAE)/100 gdw respectively. The plant extracts demonstrated significant xanthine oxidase inhibitory activity at 100 g/mL and revealed an inhibition greater than 50 % and IC_50_ values below the standard. This effect was almost similar to the activity of allopurinol (Standard drug) against xanthine oxidase (90.2 ± 0.4 %). These plant root extract will be subjected for further extensive studies to isolate and identify their active constituents which are useful for against inflammation and gout.

## Introduction

The plant extracts or their secondary metabolites have served as antioxidants in phytotherapeutic medicines to protect against various diseases for centuries related to oxidative stress and free radicals (1). Free radicals, mainly includes reactive oxygen species (ROS) such as, hydroxyl radicals, peroxyl radicals, super oxide radicals, hydrogen peroxide, singlet oxygen, and various lipid peroxides (2). ROS are also capable of reacting with membrane lipids, proteins, nucleic acids, various metabolic enzymes, and small molecules of living systems. They play an important role in the initiation and progression of various diseases such as atherosclerosis, cardiovascular diseases, aging, respiratory diseases, cancer, and gout (3). Gout develops due to the deposition of uric acid in the form of urate monohydrate crystals in the synovial joints during purine catabolism by xanthine oxidase (4). Xanthine oxidase (XO) catalyses the metabolism of hypoxanthine to xanthine, and xanthine into uric acid, which is responsible for the medical condition leading to painful inflammation called gout (5). XO also serves as an important biological source of oxygen-derived free radicals that contribute to oxidative damage to living tissues involved in many pathological processes such as inflammation, atherosclerosis, cancer and aging. *In-vitro* bioassays are used to examine test material for XO inhibition, as inhibitors of XO may be potentially useful for the treatment of gout or other XO induced diseases (6). The extraction and characterization of active compounds from medicinal plants have resulted in the discovery of new drugs, with high therapeutic value (7). The use of medicinal plants (herbs) has long history throughout the world and herbal preparations, including herbal extracts, can be found in the pharmacopoeias of numerous countries (8). A key factor in the widespread acceptance of natural or alternative therapies by the international community involves the modernization, standardization and quality control of these herbal plants, by use of modern science and technology. However, quality-related problems (lack of consistency, safety, and efficacy) seem to be overshadowing the potential genuine health benefits of various herbal products, and a major cause of these problems seems to be related to the lack of simple and reliable analytical techniques and methodologies for the chemical analysis of herbal materials (9). Modern high-performance TLC (HPTLC) is an efficient instrumental analysis, and optimised quantitative HPTLC using a densitometric evaluation can produce results analogous to those obtained with gas chromatography (GC) and high performance liquid chromatography (HPLC) (10,11). Thus, HPTLC ‘fingerprint analysis’ may be a powerful tool for the quality control of raw plant material and may be an alternative technique, particularly in the analysis of crude plant extracts. An improvement over conventional TLC, HPTLC is an instrumental technique where by special plates and instrumental resources for sampling are used and the quantitative evaluation of separations is aided by densitometry (12). The objective of this work was to describe and develop HPTLC analysis (fingerprint and densitometry) for the determination of flavonoids in plant extracts (*Asparagus racemosus, Withania somnifera, Vitex negundo, Plumbago zeylanica, Butea monosperma *and *Tephrosia purpurea*), and to determine the validity of plant remedies used for gout by examining their antioxidant and xanthine oxidase inhibitory activity.

## Experimental

*Reagents, samples and standards*

Flavonoids Standards (Quercetin, Rutin, Luteolin and Vitexin), DPPH(1,1-Diphenyl-2-picrylhydrazyl radical), 2,2-azinobis- (3-ethylbenzthiazoline-6-sulfonic acid) were purchased from Sigma–Aldrich (Mumbai-India). Trolox (6-hydroxy-2, 5, 7, 8-tetramethylchroman-2- carboxylic acid), sodium bisulfite and formic acid were from Himedia AG (Mumbai-India). Xanthine oxidase (source: microorganisms), xanthine and Allopurinol was obtained from Himedia Laboratories Pvt. *Ltd*., Mumbai, India. All other chemicals used in the study were obtained commercially and were of analytical grade.

*Plant materials*

The plants *Asparagus racemosus, Withania somnifera, Vitex negundo, Plumbago zeylanica, Butea monosperma *and *Tephrosia purpurea* were collected during the period of July to November, 2011 from local forest Nanded, India and botanically authenticated by Dr. C.N.Khobragade, School of Life Sciences, SRTM University, Nanded and deposited in department. The roots were separated, air dried over 4 days in shade and used for further analysis.

*Extraction of plant materials*

1 Kg of each plant roots were shade dried for a week, grinded by using mortar and pestle. The finely powdered samples were extracted with water, methanol: water (1:1, v:v) mixture, methanol and ethyl acetate using a mechanical shaker and Soxhlet apparatus for 4 hours. The resultant extracts were placed in an ultrasonic bath (Sonorex, model RK 512 H, Badelin, Germany) at 60 °C for 15 min and then centrifuged at 4000 rpm for 5 min (BioEra Instruments, Mumbai). 

*Animals*

Sprague–Dawley rats (150–175 g) were procured from the animal house, Maharashtra Institute of Pharmacy, Pune. They were kept in the departmental animal house at 26 ± 2 °C and relative humidity 44–56%, light and dark cycles of 10 and 14 h respectively for one week before and during the experiments. Animals were provided with standard rodent pellet diet (Amrut, India) and the food was withdrawn 18–24 h, before the experiment though water was allowed ad libitum. All studies were performed in accordance with the guidelines for the care and use of laboratory animals, as adopted and promulgated by the Institutional Animal Ethical Committee, MIP/IAEC, India (Reg. No. MIP/IAC/09-10/M1/004).

*High Performance Thin-layer chromatography (HPTLC)*

HPTLC was performed on silica gel 60 f _254,_ 20X10 cm HPTLC plates (Merck, Germany-#5642), with ethyl acetate: methanol: formic acid: water [20:2.5:0.5:2 (v/v)] as a mobile phase. The standard (Quercetin, Rutin, Luteolin and Vitexin) solutions (5.0 µL of each concentration 1 mg/mL) were applied to the plates as 10 mm bands, sample application with CAMAG-Linomat IV automated spray on band applicator equipped with a 100 µL syringe and operated with following settings: band length 10 mm, application rate 10 sec/ µL, distance between 4 mm, distance from the plate side edge1.5 cm and distance from the bottom of the plate 2 cm (10, 14). CAMAG TLC Scanner 3 was used to densitometrically to quantify the bands using WIN CATS software (Version 4 X). The scanner operating parameters were: (Mode: absorption / reflection; Slit dimension; 5 x 0.1 mm; scanning rate: 20 mm/s and monochromator band width: 20 nm at an optimized wavelength 254, 366 nm and in visible range). 

*Total polyphenol content*

Total polyphenol content was measured using Folin Ciocalteu colorimetric method described previously by Gao *et al*., (2000) (15). Quantification was done with respect to the standard curve of gallic acid. The results were expressed as gallic acid equivalents (GAE), mg/100g of dry weight (dw). All determinations were performed in triplicate (n = 3).

*Total flavonoid content.*

Flavonoids were quantified using aluminium chloride reagent. 1 mg/mL of root extract samples was dissolved in methanol, 1 mL of AlCl_3_ (2%) in methanol was added, and after incubation for 10 min, the absorbance was measured at 430 nm. The analyses were replicated (n = 3), and the contents given as mean values, plus or minus the standard deviation. Flavonoids were measured as quercetin equivalents and expressed as milligrams of each compound per 100 g of dry weight (dw) of plant root extract (16). 

*Ferric reducing/antioxidant power (FRAP) assay*

Total antioxidant potential of a sample was determined using the ferric reducing ability of plasma (FRAP assay) as a measure of antioxidant power. The assay was based on the reducing power of a compound (antioxidant). A potential antioxidant will reduce the ferric ion (Fe^3+^) to the ferrous ion (Fe^2+^); the latter forms a blue complex (Fe^2+^/TPTZ), which increases the absorption at 593 nm. Briefly, the FRAP reagent was prepared by mixing acetate buffer (300 lM, pH 3.6), a solution of 10 µM TPTZ in 40 µM HCl, and 20 µM FeCl_3_ at 10:1:1 (v/v/v). The reagent (300 µL) and plant root extracts (100 µg/mL) were added to each well and mixed thoroughly. The absorbance was taken at 593 nm after 10 min. Standard curve was prepared using different concentrations of trolox. All solutions were used on the day of preparation. The results were corrected for dilution (*e.g*. to 1000 mL) and expressed in l M trolox per 100 g dry weight (dw). All determinations were performed in triplicates (17).

*DPPH antioxidant assay*

DPPH radical-scavenging activity was determined using the method. DPPH (100 µM) was dissolved in pure ethanol (96%). The radical stock solution was prepared fresh daily. The DPPH solution (1 mL) was added to 1 mg/mL of root extracts with 3 mL of ethanol. The mixture was shaken vigorously and allowed to stand at room temperature in the dark for 10 min. The decrease in absorbance of the resulting solution was monitored at 517 nm at 10 min. The results were corrected for dilution and expressed in µM trolox per 100 g dry weight (dw). All determinations were performed in triplicate (18).

*ABTS antioxidant assay*

The free radical-scavenging activity was determined by ABTS radical cation decolorization assay. ABTS was dissolved in water to a 7 µM concentration. ABTS radical cation (ABTS^.+^) was produced by reacting ABTS stock solution with 2.45 µM potassium persulfate (final concentration) and kept in the dark at room temperature for 12–16 h, before use. The radical was stable in this form for more than two days, when stored in the dark at room temperature. For the study of infusion, the samples containing the ABTS^.+^ solution were diluted with redistilled water to an absorbance of 0.7 (±0.02) at 734 nm and equilibrated at 30 ^0^C. A reagent blank reading was taken (*A*_0_). After addition of 3.0 mL of diluted ABTS^.+^ solution (*A*_734nm_ = 0.7±0.02) to 30 µg/mL of root extracts, the absorbance reading was exactly 6 min after initial mixing (*A*_t_). The results were corrected for dilution and expressed in µM trolox per 100 g dry weight (dw). All determinations were performed in triplicate (19). Rats were grouped into four groups (six animals in each group). The first group served as normal control and received 1% CMC (10 mL/Kg b.wt/day, p.o.). The second group served as negative control (CCl_4_ treated). The third group supplemented with each plant root extracts (50 and 100 mg/Kg b.wt/day respectively, p.o.) for 14 days. Fourth group was treated with standard vitamin E orally for 14 days at a dose of 50 mg/Kg b.wt/day (p.o.). The animals of all groups except first were administered simultaneously CCl_4_: liquid paraffin (1:1, 2 mL/Kg b.wt/day, s.c.) on alternate days after 30 min of administration of the each plant extracts and vitamin E. Twenty four hours after the last dose of CCl_4_ animals were sacrificed. Liver was dissected out from each animal and used for biochemical investigations. Liver homogenate 10.0% (w/v) was prepared with 0.15 M KCl and centrifuged at 12,000 rpm for 15 min. The homogenate supernatant was used for the biochemical estimation. LPO was estimated by standard method of Ohkawa *et al*., (1979) and results are expressed as nmole of MDA formed/mg protein (20). Superoxide dismutase (SOD) activity was estimated by the inhibition of nicotinamide adenine dinucleotide (reduced)- phenazine methosulphate–nitro blue tetrazolium reaction system as adapted by Kakkar *et al*., (1984) (21). The results have been expressed as units (U) of SOD activity/mg protein. Catalase was estimated by following the breakdown of H_2_O_2_ and expressed as l mole of H_2_O_2_ consumed/mg protein (22). Glutathione (GSH) level was determined according to the method of Ellmann (1959) (23).

*In-vitro xanthine oxidase inhibitory activity*

The XO activity was assayed spectrophotometrically under aerobic conditions using plant extracts. The assay mixture consist of 1 mL of root extract solution of each plant, and standard drug allopurinol (25, 50, 75, and 100 µg/mL), 2.9 mL of phosphate buffer (pH 7.5), and 0.1 mL of enzyme solution (0.01 units/mL in phosphate buffer, pH 7.5) was added, which was prepared immediately before use. After pre incubation at 25 ^0^C for 15 min, the reaction was initiated by the addition of 2 mL of substrate solution (150 mM xanthine in the same buffer). The assay mixture was incubated at 25 ^0^C for 30 min. The reaction was then stopped by the addition of 1ml of 1N hydrochloric acid, and the absorbance was measured at 290 nm using a UV spectrophotometer. A blank was also prepared in the same way, but the enzyme solution added to the assay mixture after adding 1N hydrochloric acid. The assay was done in triplicate. One unit of XO is defined as the amount of enzyme required to produce 1 mmol of uric acid per min at 25 ^0^C. XO activity was expressed as the percentage inhibition of XO in the above assay system, calculated as, % Inhibition = (*A *− *B*) − (*C *− *D*) *A *− *B *× 100 

Where *A *is the activity of the enzyme without test extract, *B *the control of *A *without test extract and enzyme, *C *and *D *are the activities of the test extract with and without XO. Allopurinol (10, 25, 50 and 100 µg/mL), a known inhibitor of XO, was used as a positive control. IC_50_ values were calculated from the mean values of data (24).

*Statistical analysis*

Values were represented as mean±S.D and data were analyzed using ANOVA followed by Dunnett’s test. *P *< 0.001 was considered significant.

## Results and Discussion

The main objective of the study is to identify and quantify flavonoids, which have been shown to be among the active principles in the studied plants. For this purpose, a novel method called HPTLC, were described for extraction and identification of flavonoids from complex plant root extracts. The resultant extracts, were then filtered concentrated under reduced pressure and evaporated to dryness and the percentage yield were recorded in Table1 and used for the antioxidant and xanthine oxidase inhibitory activity (13). 

**Table 1:** Percent yield of plant extract after extraction (mg mL^-1^)^a)^

**Plant codes**	**Taxa**	**Family**	**% yield (g)**
P1	*A. racemosus*	*Asparagaceae*	4.0 ± 0.28
P2	*W. somnifera*	*Solanaceae*	6.2 ± 0.18
P3	*V. negundo*	*Lamiaceae*	8.1± 0.32
P4	*P. zylenica*	*Verbenaceae*	5.6± 0.58
P5	*B. monosperma*	*Fabaceae*	7.2 ± 0.64
P6	*T. purpurea*	*Acanthaceae*	6.5± 0.42

a ^)^mean ± SD (n=3)

*HPTLC fingerprint analysis*

**Figure 1:**
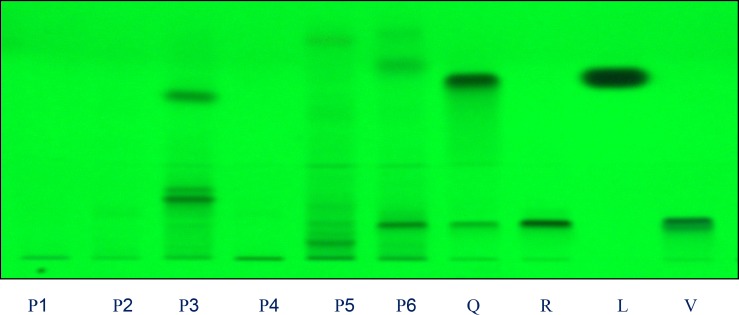
Chromatograms obtained from separation of plant extracts of P1, P2, P3, P4, P5, P6 and standards Q: quercetin, R: rutin, C: Luteolin and V: vitexin (sample codes are explained in Table 1) Visualization was under UV light of wavelength 254 nm

**Figure 2:**
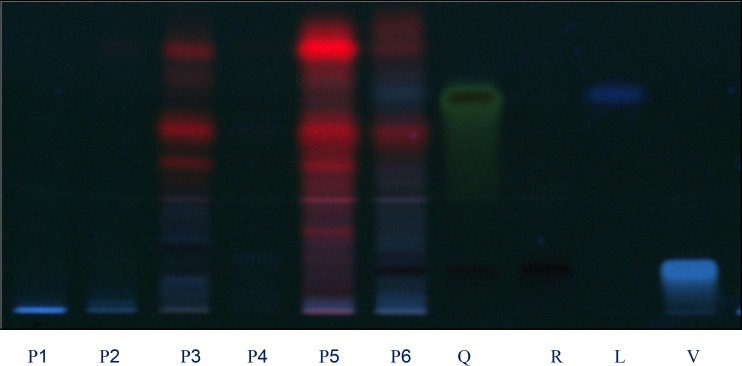
Chromatograms obtained from separation of plant extracts of P1, P2, P3, P4, P5, P6 and standards Q: quercetin, R: rutin, C: Luteolin and V: vitexin (sample codes are explained in Table 1) Visualization was under UV light of wavelength 366 nm

Four different mobile phases previously described for the separation of flavonoids were tested, using silica gel HPTLC plates, namely ethyl acetate: formic acid: water (6:1:1, v/v) (25), ethyl acetate: formic acid: acetic acid: water (100:11:11:26, v/v) (10), ethyl acetate: methyl ethyl ketone: formic acid: water (50:30:10:10, v/v) (9), and ethyl acetate: formic acid: water (82:9:9, v/v) (26). The only phase that allowed us to visualize differences among the extracts studied was the newly developed mobile phase ethyl acetate: methanol: formic acid: water (20:2.7:0.5:02 v/v). The use of these solvent systems provides good separation of the flavonoids with respective to *F*ro (Figures 1 and 2). This mobile phase offers an improvement over the earlier method described Brasseur and Angenot and may replace the one described in the last edition of the *European Pharmacopoeia *(9,25), Quercetin, Rutin, Luteolin and Vitexin (*R*f = 0.97, 0.53, 0.59 and 0.78, respectively were found in all the species studied. Of the compound available as standards Rutin was present in most of the plants species. The flavonoids content found in these plants show the respective order as Rutin > Luteolin > Vitexin > Quercetin. The florescence bands of most of the flavonoids are not visible at 254 nm wavelength but they are visible at 366 nm.

*Determination of phenolic and flavonoids content*

The amount of total phenolics, measured by Folin–Ciocalteu method, varied widely in plant root materials and ranged from 10.21 to 28.17 mg GAE/100 g dry weight (dw). The highest level of phenolics was found in *A.racemosus,* while the lowest was in* P. zylenica*.* B.monosperma *and *W. somnifera* root extract*, *(24.0 mg GAE/100 g dw) also had very high levels of phenolics. Other herbs with high levels of phenolics i.e. *T. purpurea* (18.02 mg GAE/100 g dw) and *V. negundo* (12.45 mg GAE/100 g dw) root respectively (Table 2). 

**Table 2:** *In* *-* *vitro* antioxidant capacity, total phenolic and flavonoid content in selected plant^a^.

Scientific Name	Total phenolic content^b^	Total flavonoid content^c^	TEAC ( µM trolox/100 g dw)		
			ABTS	DPPH	FRAP
*A.racemosus*	28.17 ± 0.87	10.10 ± 2.14	55.80 ± 1.23	180 ± 2.56	380 ± 4.85
*W. somnifera*	24.02 ± 0.67	09.12 ± 4.34	50.70 ± 2.05	360 ± 5.46	560 ± 5.12
*V. negundo*	12.45 ± 0.65	06.12 ± 3.12	140.00 ± 3.01	253 ± 3.56	280 ± 2.10
*P. zylenica*	10.21 ± 0.42	05.80 ± 1.23	70.50 ± 2.09	430 ± 5.22	350 ± 4.34
*B.monosperma*	24.07 ± 3.20	08.24 ± 1.45	57.80 ± 1.23	085 ± 0.56	260 ± 3.80
*T. purpurea*	18.02 ± 0.67	06.80 ± 5.10	45.80 ± 3.04	140 ± 2.34	185 ± 3.21

a All values are the means of three measurements.

b Total phenolic content expressed as mg of GAE/100 g of dry weight (dw).

C Total flavonoid content expressed as mg of GAE/100 g of dry weight (dw).

*In-vitro antioxidant activity*

Among the six medicinal plant tested in this study, all plant root extracts revealed excellent to significant antioxidant activities by ABTS, DPPH and FRAP assay methods (Table 2). The results obtained in the present study showed that, the medicinal plant were relatively high but not very high in polyphenols. Total phenolic contents of the six studied plants revealed the following order: *A.racemosus, *> *B.monosperma *> *W. somnifera* > *T. purpurea* > *V. negundo* > *P. zylenica*. Significant differences between the results were likely due to genotopic and environmental differences (namely, climate, location, temperature, fertility, diseases and pest exposure) within species, choice of parts tested, time of taking samples and determination methods (27). In general, antioxidant activity of flavonoids depends on the structure and substitution pattern of hydroxyl groups. The essential requirement for effective radical scavenging is the 3, 4 orthodihydroxy configuration in ring B and 4- carbonyl group in ring C. The presence of 3-OH group and 5-OH groups, giving a catechol-like structure in ring C, is also beneficial for the antioxidant activity of flavonoids. The presence of the C2–C3 double bond configured with a 4-keto arrangement is known to be responsible for electron delocalization from ring B and it increases the radical-scavenging activity. In the absence of the o-dihydroxy structure in ring B, a catechol structure in ring A can compensate for flavonoid antioxidant activity. The relationship between the chemical structure of flavonoids and their radical-scavenging activities was analyzed by Bors, *et al.* (28). Flavonols (quercetin, myricetin, kaempferol and isorhamnetin) have a hydroxyl group at position 3, which suggests a structurally important role of the 3 OH group of the chroman ring responsible for enhancement of antioxidant activity (25). In our research, the plants might be with high contents of quercetin, kaempferol and luteolin showing high antioxidant activity. In addition, a significant linear relationship was found between the antioxidant activity, especially with ABTS and FRAP, while phenolic compounds were major contributors to antioxidant activity. 

*In-vivo antioxidant activity*

Administration of CCl_4_ significantly increased the levels of LPO (P < 0.001) with significant decrease in GSH content (P < 0.001). The activities of antioxidant enzymes SOD (P < 0.001) and CAT (P < 0.001) were reduced in both liver and kidney of CCl_4_ induced toxic rats (Table 3). Co-treatment of rats with plant extract at a dose of 50 mg/Kg and 100 mg/Kg/day, for 14 days markedly prevented these CCl_4_ induced alterations and maintained enzyme levels near to normal values. Vitamin E also significantly inhibited the elevated levels of LPO and increased the levels of GSH, SOD and CAT in CCl_4_ induced toxic rats (P < 0.001) (Table 3). In CC1_4_ induced toxicity, the initial reaction is a reduction and homolytic cleavage of CC1_4_ to the trichloromethyl radical (CC1_3_). This radical may react directly with cellular macromolecules or may react with oxygen to form the trichloromethylperoxyl radical (^.^OOCC1_3_), which may then attack lipids more readily than CC1_3_ (29). Plant extract treatment reduced the elevated level of lipid peroxidation and increased the depleted levels of cellular GSH significantly in CCl_4_ induced toxic rats. The plant extracts also restored the levels of antioxidant enzymes such as SOD and CAT almost back to the normal levels. SOD plays an important role in the elimination of ROS and protects cells against the deleterious effects of super oxide anion derived from the peroxidative process in liver and kidney tissues (30), and the observed increase in SOD activity suggests that the plant extracts has an efficient protective mechanism in response to ROS. CAT considered as most important H_2_O_2_ removing enzyme and also a key component of antioxidative defense system (31). Here CAT activity was increased and then restored to normal levels on administration of plant extracts. The present study demonstrates that the plant extract possesses high phenolic content and antioxidant activity. 

*Xanthine oxidase inhibition*

The* in-vitro *xanthine oxidase inhibition of studied plant root extract revealed significant inhibitory activity at 100 µg/mL, among which *A.racemosus* and *T. purpurea* (55.56%) showed an inhibition greater than 50% followed by *W. somnifera, V. negundo, B.monosperma and P. zylenica* (72.22%) were found to be active at a concentration of 50 µg/ml, among the studied plants *A.racemosus* and *T. purpurea* showed significant inhibition towards xanthine oxidase. In general, the methanolic extracts were found to be more active than the aqueous extracts. All the plants *i.e*. *A.racemosus* , *T. purpurea*, *W. somnifera, V. negundo, B.monosperma and P. zylenica* extracts produced significant activity up to 100 µg/mL concentrations for the inhibition of XO activity. The results were compared with the standard drug allopurinol, which showed 90.2% inhibition at 100 µg/mL concentration with IC_50_ value 6.75 µg/mL (Table 4). 

**Table 3:** *In* *-* *vivo* antioxidant/radical scavenging activity of ethyl acetate fraction of plant extracts^a^.

**Groups and doses**	**Extract**	**Liver**				**Kideney**			
		**GSH**	**LPO**	**SOD**	**Catalase**	**GSH**	**LPO**	**SOD**	**Catalase**
Normal Control**		0.65 ± 0.02	35.2 ± 0.8	96.6 ± 2.6	42.1 ± 0.4	0.65 ± 0.04	26.2 ± 3.1	120.1 ± 7.2	45.2 ± 2.5
Negative Control**		5.23 ± 0.01	16.2 ± 2.4	55.8 ± 1.8	280.2 ± 0.6	3.10 ± 0.50	18.2 ± 3.2	70.1 ± 3.5	22.2 ± 2.1
Plant extracts**	P1	4.12 ± 0.42	25.5 ± 1.2	100.2 ± 3.2	30.5 ± 1.5	2.58 ± 0.25	24.2 ± 4.5	100.2 ± 3.7	40.2 ± 1.8
	P2	3.21 ± 0.15	20.4 ± 3.2	90.1 ± 2.2	28.8 ± 3.2	1.25 ± 0.12	20.2 ± 3.2	94.8 ± 1.8	34.1 ± 4.2
	P3	1.12 ± 0.22	15.5 ± 0.9	75.3 ± 1.8	19.5 ± 1.8	1.00 ± 0.34	15.6 ± 1.8	90.5 ± 2.0	31.2 ± 2.0
	P4	2.01 ± 0.30	18.6 ± 1.5	88.2 ± 5.2	23.5± 1.2	1.12 ± 0.08	18.1 ± 2.1	74.7 ± 2.5	33.5 ± 1.1
	P5	3.45 ± 0.50	22.5 ± 2.5	98.1 ± 3.2	25.2 ± 2.2	2.00 ±0.32	22.4 ± 1.4	95.4 ± 3.0	36.1 ± 1.5
	P6	1.15 ± 0.20	15.2 ± 1.4	60.1 ± 1.5	15.8 ± 2.8	0.98 ± 0.11	12.4 ± 3.5	70.3 ± 2.5	20.2 ± 3.4
Standard**		0.68 ± 0.02	35.2 ± 1.5	112.2 ± 5.2	40.5 ± 2.5	0.95 ± 0.12	30.2 ± 1.5	130.1 ± 3.6	40.1 ± 2.4

Results are expressed as: LPO: nmole MDA/mg protein; GSH: µg/mg protein; SOD: U/mg protein; CAT: U/mg protein.

* Values are mean ± SEM for six rats.

a P < 0.001 considered as significant.

** Normal Control: (1% CMC, 10 mL/Kg), Negative Control: CCl4 treated (0.5 mL/Kg), Plant extracts: CCl4 (50 mg/Kg + 0.5 mL/Kg) Standard: (Vitamin E + CCl4 (50 mg/Kg + 0.5 mL/Kg).

**Table 4:** *In* *-* *vitro* xanthine oxidase inhibitory activity of plant extracts*.

**Species/family**	**Extract used**	**Percentage xanthine oxidase inhibition**				**IC**_50 _ **(µg/mL)**
		10 µg/mL	25 µg/mL	50 µg/mL	100 µg/mL	
*A.racemosus*	Aqueous	18.4 ± 0.2	28.3 ± 0.1	42.8 ± 0.4	68.8 ± 0.2	4
	Methanolic	38.4 ± 0.2	48.2 ± 0.6	62.1 ± 0.2	80.0 ± 0.5	5
*W. somnifera*	Aqueous	15.2 ± 0.2	22.5 ± 0.5	36.8 ± 0.1	65.8 ± 0.4	3
	Methanolic	35.6 ± 0.1	42.4 ± 0.3	55.6 ± 0.2	76.0 ± 0.5	5
*V. negundo*	Aqueous	10.4 ± 0.2	22.5 ± 0.1	38.8 ± 0.4	60.8 ± 0.2	5
	Methanolic	15.6 ± 0.2	28.2 ± 0.6	45.8 ± 0.2	70.0 ± 0.5	6
*P. zylenica*	Aqueous	24.2 ± 0.1	32.1 ± 0.1	45.6 ± 0.5	60.1 ± 0.4	4
	Methanolic	26.1 ± 0.2	35.0 ± 0.3	48.2 ± 0.2	65.4 ± 0.1	5
*B.monosperma*	Aqueous	30.0 ± 0.1	36.5 ± 0.3	42.2 ± 0.5	65.5 ± 0.6	5
	Methanolic	34.6 ± 0.1	38.2 ± 0.6	50.6 ± 0.1	75.0 ± 0.5	5
*T. purpurea*	Aqueous	24.0 ± 0.2	42.5 ± 0.1	54.8 ± 0.1	70.5 ± 0.1	4
	Methanolic	25.6 ± 0.2	45.2 ± 0.3	58.0 ± 0.7	80.0 ± 0.2	6
Allopurinol		25.5 ± 0.2	30.8 ± 0.1	42.0 ± 0.2	50.2 ± 0.4	7

* Values are Mean± S.D. of three parallel measurements.
